# Application of Halloysite Nanotubes in Cancer Therapy—A Review

**DOI:** 10.3390/ma14112943

**Published:** 2021-05-29

**Authors:** Anna Karewicz, Adrianna Machowska, Martyna Kasprzyk, Gabriela Ledwójcik

**Affiliations:** Department of Chemistry, Jagiellonian University in Cracow, Gronostajowa 2, 30-387 Cracow, Poland; adrianna.machowska@doctoral.uj.edu.pl (A.M.); martyna.kasprzyk@student.uj.edu.pl (M.K.); gabriela.ledwojcik@student.uj.edu.pl (G.L.)

**Keywords:** halloysite nanotubes, cancer cells, controlled release, targeted delivery, nanostructured surfaces, magnetic surfaces

## Abstract

Halloysite, a nanoclay characterized by a unique, tubular structure, with oppositely charged interior and exterior, suitable, nanometric-range size, high biocompatibility, and low cost, is recently gaining more and more interest as an important and versatile component of various biomaterials and delivery systems of biomedical relevance. One of the most recent, significant, and intensely studied fields in which halloysite nanotubes (HNTs) found diverse applications is cancer therapy. Even though this particular direction is mentioned in several more general reviews, it has never so far been discussed in detail. In our review, we offer an extended survey of the literature on that particular aspect of the biomedical application of HNTs. While historical perspective is also given, our paper is focused on the most recent developments in this field, including controlled delivery and release of anticancer agents and nucleic acids by HNT-based systems, targeting cancer cells using HNT as a carrier, and the capture and analysis of circulating tumor cells (CTCs) with nanostructured or magnetic HNT surfaces. The overview of the most up-to-date knowledge on the HNT interactions with cancer cells is also given.

## 1. Introduction

Cancer is one of the deadliest diseases worldwide, causing 7.6 million deaths a year globally. Despite growing social awareness, various medical screening programs, and other efforts to reduce the risk of cancer, the disease is most often detected only at its late stages [1]. That is because the initial symptoms of cancer are often difficult to notice and rather unspecific, leading to late diagnosis, resulting in a worse prognosis for the patient. Due to the enormous mortality rate, a large amount of research is currently carried out on the various modern methods of cancer detection and treatment. Various delivery systems are proposed in order to deliver chemotherapeutics to tumor tissue effectively and safely. Among them, the silicon-based nanoparticles and other delivery systems found an important place [2], mainly due to the silicon and carbon similarities and relatively high biocompatibility [3,4]. Mesoporous silica nanoparticles (MSNs) are a relatively new carrier, proposed for drug delivery applications, but already gaining substantial interest due to their unique properties, mainly the possibility to fine-tune their size, as well as the pore size, their volume, and structure [5]. Various MSN-based drug nanocarriers for anticancer treatment were reported [6], including stimuli-responsive formulations [7,8] and systems for targeted cancer therapy [9,10]. Keshavarz et al. [11] have recently proposed a new material composed of silica nanoparticles self-assembled into the 3D nanoweb, which was able to induce selective apoptosis in HeLa cancer cells. The same group has reported on the photoluminescent silicon nanoprobe characterized by excellent optical properties (exceeding those of the quantum dots), which was also selectively taken up by the cancer cells [12]. A significant part of the research focuses on the use of natural materials due to their centuries-old healing properties, broad availability, often combined with low toxicity, and high biocompatibility [13,14]. Nanoclays also belong to this class of materials [15,16,17]. They have a layered, crystalline structure made of hydrated aluminosilicates. The layers are composed of silicate tetrahedrons made by the Si^4+^ centers surrounded by four oxygen atoms, and aluminum hydroxide octahedrons with the Al^3+^ ion as the center, surrounded by six hydroxyl groups. The arrangement of these layers allows dividing nanoclays into various classes of minerals, such as hectorite, bentonite, kaolinite montmorillonite, and halloysite [18]. Various nanoclays have been applied with success as carriers in the delivery of anticancer drugs [19]. Kaolin-based systems for doxorubicin (DOX) delivery were studied by Zhong et al. [20], and the influence of the intercalated organic guest molecules of different sizes on the release kinetics of the drug was confirmed. Further, 5-fluorouracil was also loaded into the methoxy-modified kaolinite [21]. Authors showed that the functionalization allowed increasing drug loading significantly and that the interlayer-loaded drug was in a more thermally stable, amorphous state. Montmorillonite was combined with alginate and carboplatin to form a hybrid delivery system [22]. The obtained material allowed for the effective, oral administration of the drug, offering an attractive alternative to the intravenous route. Montmorillonite was also combined with PLGA, loaded with paclitaxel, and decorated with trastuzumab for targeted delivery to breast cancer [23]. Poly(lactide)-vitamin E derivative/montmorillonite nanoparticles loaded with docetaxel showed a two- to fourfold higher toxic effect to cancer cells and more than 25 times higher half-life, compared to the widely used clinical dosage for this drug (Taxotere^®^) [24].

Among the nanoclays, halloysite is proposed most often in the anticancer applications, mainly due to its unique structure, ability to load drugs via either adsorption [25] or intercalation [26], as well as because of its ability to provide the tunable release [27] and the rare possibility to accommodate simultaneously two different drugs with dissimilar physicochemical properties [28]. The nanotubular structure of halloysite allows the encapsulation of a vast variety of drugs [29,30,31]. The outer surface of halloysite nanotubes (HNTs) may be easily modified in order to adjust the properties crucial for a carrier (charge, polarity) [32] to introduce biologically relevant molecules (ligands, antibodies) [33,34] or to attach larger molecules/nanoparticles (e.g., magnetic particles, quantum dots) [35,36]. This enables the use of halloysite nanotubes (HNTs) in a wide range of anticancer therapies that involve prolonged, controlled, and/or targeted delivery of cytostatics or other antitumor agents, as well as the effective capture of cancer cells using nanostructured or magnetic surfaces [19,37]. Last, but not least, HNTs are biocompatible, easily available, cost effective, and may be considered to be a “green” material [38]. In comparison to MSNs, the main advantages of HNTs are the possibility to encapsulate and protect the larger molecules (e.g., proteins) inside the HNTs’ lumen, and the possibility to entrap more than one drug simultaneously: in the lumen and by intercalation between the layers of the nanotube wall. The main HNTs’ advantage over the other nanoclays lies in its tubular structure and the opportunities it creates for drug loading and controlled release.

The studies on the application of HNTs in cancer diagnosis and treatment are relatively new. Halloysite was used to deliver hydrophobic molecules only 20 years ago by Price et al. [39] while the first report on HNTs as a carrier in prolonged drug delivery was published by Levis and Deasy [40] only in 2003. Almost a decade later, HNTs were used in anticancer research: initially, in 2010 by Hughes et al. to form nanostructured surfaces for cancer cell capture [41], and then in 2012 by Guo [42] and Vergaro [43] as a nanovehicle for anticancer drugs. In the last 10 years, HNTs became more and more intensely studied as a component of the anticancer therapies with ca. 60 papers published, covering controlled and targeted delivery, including gene and siRNA delivery, as well as using nanostructured, ligand functionalized, and magnetic surfaces to capture cancer cells. This review summarizes the state of art in this new, exciting field while also providing some insight into the current knowledge about the HNTs’ cytocompatibility and interactions with cancer cells.

## 2. HNTs in Controlled Delivery of Anticancer Drugs

Due to its unique nanotubular structure, HNTs are often loaded with biologically active compounds that can then be released in a prolonged manner. Table 1 summarizes the literature reports on the chemotherapeutics successfully entrapped in HNTs.

Among many anticancer agents, those used most frequently in a combination with HNTs are doxorubicin (DOX) and curcumin (CUR). Vergaro et al. [43] were first to propose the use of HNTs in the controlled delivery of resveratrol, an anticancer agent, a natural, polyphenolic compound that can be found, e.g., in red grapes and peanuts. Resveratrol can function as an ERα receptor ligand and shows antiproliferative and proapoptotic activity in ERα positive human breast cancer cell line MCF-7. The authors used HNTs as a carrier for resveratrol and coated drug-containing HNTs with polymeric multilayers composed of protamine and dextran sulfate sodium salt using the layer-by-layer (LbL) technique in order to control the release rate of the active agent. Due to the presence of proteolytic enzymes in the MCF-7 culture, the multilayer coating was gradually degraded, and resveratrol was slowly released. The enhanced anticancer effect, most probably related to the selective interaction of the drug with the ERα receptor, was confirmed. Yang and et al. [44] described the loading of a cytostatic drug, doxorubicin (DOX), into the HNTs grafted with chitosan oligosaccharides (HNTs-g-COS) as another promising system for effective treatment of breast cancer. The authors applied oligomers, rather than higher molecular weight chitosan, based on the previous studies showing that the latter decreases the loading efficiency and hinders the cellular uptake. Compared to uncoated HNTs, the resulting HNTs-g-COS structure had a positive surface charge, improved cytocompatibility, and decreased hemolysis ratio. The in vitro release studies performed in PBS (pH = 7.4) and cell lysate (resembling tumor environment) confirmed that DOX was released in a controlled manner in both media but much faster (61.9% of DOX released after 12 h) in the lysate. Based on the results of the flow cytometry, it was found that the system had an apoptotic effect on the MCF-7 breast cancer cell line. HNTs-g-COS, loaded with DOX, was retained in both cytoplasm and nucleus of the cancer cell for a long time and induced apoptosis by a combined mechanism including overproduction of reactive oxygen species (ROS) and mitochondrial damage. It was also shown to produce more ROS than free DOX and to attack the nucleus. Additionally, in vivo antitumor experiments showed that DOX-containing HNTs-g-COS had a better tumor inhibition efficacy, compared to DOX, and, at the same time, was not toxic to lung, heart, liver, or kidney tissues.

Another system to deliver DOX to breast cancer cells was proposed by Wu et al. in 2018 [33]. Halloysite nanotubes were decorated with polyethylene glycol (PEG) chains, and then folic acid was conjugated to PEG to actively target cancer, forming an HNTs-PEG-FA system. In agreement with the previous findings, the developed system induced apoptosis of MCF-7 cells and significantly inhibited their proliferation. Additionally, the system was shown to be specifically targeting cells expressing the folic acid receptor (FR+). DOX loaded in the tested system showed an increased ability to generate ROS in the MCF-7 cells in comparison to the free drug. DOX loaded into HNTs-PEG-FA was released slowly, up to 35 h. The proposed system was shown to be less cytotoxic to the heart, compared to free DOX, and thus was considered to be a very good candidate for breast cancer therapy [33].

**Figure 1:**
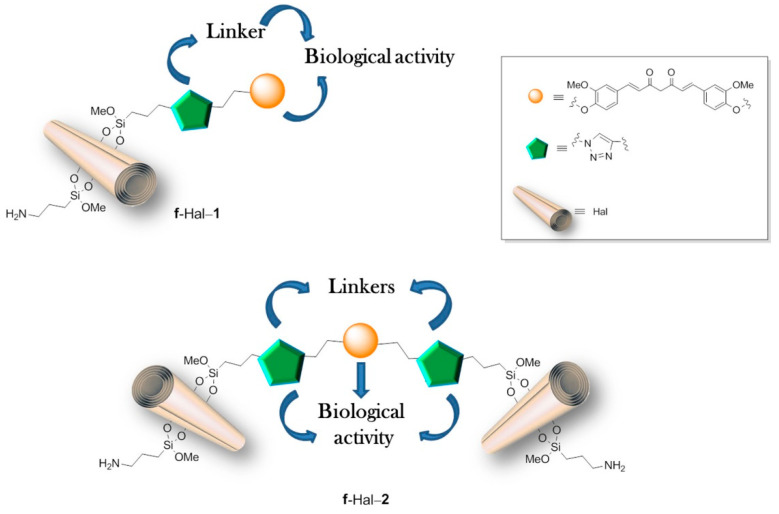
Cartoon representation of the synthetized f-Hal-1 and -2 carrier systems. Reprinted from *Applied Clay Science*, Vol 184, Marina Massaro, Paola Pomaa, Carmelo G. Colletti, Anna Barattucci, Paola M. Bonaccorsi, Giuseppe Lazzara, Giuseppe Nicotra, Filippo Parisic, Tania M.G. Salerno, Corrado Spinella, Serena Riela, Chemical and biological evaluation of crosslinked halloysite-curcumin derivatives, 105400 (Pages 1–10), Copyright (2020), with permission from Elsevier [48].

Studies on HNTs loaded with an anticancer agent were also conducted for curcumin (CUR). Massaro et al. (2020) [48] described the sophisticated HNT-based system with covalently linked curcumin derivative, having one or two terminal alkyl groups (Cur-1 and Cur-2) and further modified by introducing another CUR derivative (Cur-3) into the nanotube’s lumen, as illustrated in Figure 1. To prepare such a system HNTs surface was first modified using click chemistry (the Huisgen 1,3-dipolar cycloaddition), and then curcumin derivative Cur-1 or Cur-2 was bound to HNTs surface. Cur-2 allowed obtaining larger systems by crosslinking two different nanotubes. Hydrophobically modified Cur-3 or, alternatively, DOX was then loaded into the nanotubes. An increased loading of Cur-3 was observed in the covalently modified systems (9%) in comparison to untreated halloysite (3.6%), most probably due to the π–π interactions between aromatic rings of Cur-3 and those previously attached to the surface of HNTs. Cur-3 was almost fully retained within the modified systems up to 60 h, possibly due to these same interactions, while DOX was released almost completely within 24 h. In the final part of the study, the cytotoxic effect of multifunctional carriers was tested on two breast cancer cell lines (SUM-149 and MDA-MB-231) and two acute myeloid leukemia cell lines (HL60 and HL60R). The results showed high cytotoxic activity of the designed systems and additionally showed that the cytotoxic effect was closely correlated with their size and the release profile of the loaded drug. Dual delivery systems containing Cur-3 generally showed increased, although cell type-dependent, cytotoxicity. Experiments have also shown an enhanced antiproliferative effect, even in multidrug-resistant cancer cell lines. Further studies on the in vitro and in vivo activity are currently underway to assess the effects of the new systems on multidrug resistance.

Bulbul et al. [49] proposed a curcumin delivery system based on the fibrous electrospun PCL/PEO/HNTs membranes loaded with curcumin. To optimize the drug loading HNTs were modified with 3-aminopropyltriethoxysilane (APTES) or 3-glycidoxypropyltrimethoxysilane (GPTMS) prior to incorporating them into the membranes. PCL/PEO electrospun fibrous membranes containing both systems at the concentration of 0.3% were loaded by the addition of 3% of curcumin. The membranes with modified HNTs showed higher loading capacity than the ones containing untreated HNTs. Importantly, in vitro studies showed that the PCL/PEO-Cur/HNT-APTES membrane had the slowest release profile and the highest cytotoxicity against the MCF-7 cell line.

Anticancer properties of HNT-based systems were studied for breast cancer, as well as for colon cancer, osteosarcoma, and bladder cancer treatment. Colon cancer is a common cause of death, and the incidence of this type of cancer increases with age. Conventional drugs show limited effectiveness in its therapy, mainly due to their lack of specificity of action and systemic side effects. Therefore, the carrier-based systems allowing for controlled delivery of anticancer drugs are, in this case, very popular [53]. Rao et al. [54] have reported a new system for effective oral delivery of 5-fluorouracil (5-FU). Hydroxyethyl methacrylate (HEMA) was copolymerized with sodium hyaluronate and N,N-methylenebis(acrylamide) as a crosslinker, forming a pH-sensitive, three-dimensional hydrogel network. The drug was first incorporated in the HNTs, which were then embedded into the previously synthetized nanocomposite hydrogel. The main aim was to prepare the system with good control over the place of 5-FU delivery, with minimal release in the stomach, and prolonged, effective release in the intestine. To investigate the properties of the system, in vitro release studies were thus carried out for 2 h at a pH of 1.2, simulating gastric conditions, and then for longer times at a pH of 7.4, simulating the intestine environment. The in vitro release studies performed for the nanocomposite hydrogels with higher HNTs content (5–7.5%) gave quite promising results: only 10% of the drug was released in the stomach-simulating conditions, while a much faster yet well-controlled release was observed in the intestinal fluid for 70 h. The release was faster at the higher hyaluronate content and slower at the higher HNTs content. The obtained hydrogels were more effective in delivering 5-FU when compared to HNTs-free hydrogels containing 5-FU, as well as to the HNTs with an encapsulated drug.

Celecoxib and atorvastatin are both effective in the prevention and treatment of colon cancer but are characterized by different physicochemical properties, while showing a synergistic effect. Li et al. [50] have prepared atorvastatin-loaded HNTs encapsulated in polymeric microspheres containing celecoxib as a new, dual delivery system for colon cancer therapy. To enlarge the inner diameter of the nanotubes and thus increase the drug loading, HNTs were acid-modified prior to their loading with a drug. Atorvastatin-loaded HNTs were then encapsulated in the polymeric microspheres containing celecoxib using microfluidics. pH-responsive hydroxypropyl methylcellulose acetate succinate (HPMCAS) was used as a polymeric matrix to form microspheres. The system constructed in this way showed a spherical morphology, pH-dependent dissolution behavior, and a narrow particle size distribution. The microparticles were designed for oral administration, and they were able to retain both drugs at pH values below 6.5, protecting them from premature release in the gastrointestinal tract. The results were encouraging, and the obtained system was suggested as a versatile platform for the oral codelivery of multiple bioactive agents with significantly different physicochemical properties and synergistic effects.

The new systems based on HNTs were also recently proposed for the effective treatment of osteosarcoma. This type of cancer occurs most often in children and is difficult to treat, with surgery and chemotherapy combination as the only effective solution. Taking also into account the extremely high number of osteosarcoma relapses, the new effective agents are being intensely sought. A 2014 publication by Sun et al. [51] describes the use of halloysite as a carrier for drugs such as methotrexate, artemisinin, quercetin, and taurolidine. The inhibition of cancer cell proliferation and the ability to induce apoptosis were tested in the cultures of osteosarcoma cells (OC, UMR-106, and ATCC). For methotrexate-encapsulating HNTs, cancer cell death was shown to be rather low, even though the proliferation was significantly inhibited. However, in the case of nanotubes loaded with artemisinin, quercetin, and taurolidine, both visible apoptosis and highly inhibited proliferation were observed. All tested drugs provided strong evidence that such systems can be successfully used in the treatment of osteosarcoma.

Although controlled release can provide the chemotherapy with additional, significant advantages, in the case of cancer treatment, the significant, often severe, unwanted side effects appear due to the inevitable cytotoxicity of the drug to normal cells. One of the best approaches is targeted delivery, in which the carrier system allows to control the release rate and also directs the drug specifically and selectively to the cancer tissue. Those systems will be discussed in the next chapter.

## 3. Halloysite-Based Delivery Systems for Targeted Cancer Therapy

**Figure 2:**
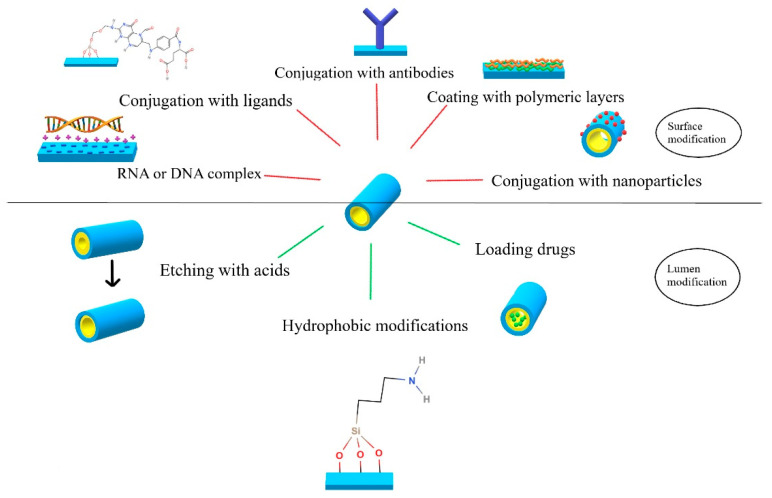
HNT modifications designed for effective delivery of anticancer drugs.

Active targeting can make a delivery of a drug significantly more efficient and safer. It is especially important in chemotherapy, where the influence of chemotherapeutics on healthy cells must be as low as possible. Hence, targeted cancer therapy using specifically modified nanocarriers is being broadly explored. The overview of the possible HNTs modifications for controlled and targeted drug delivery is presented in Figure 2.

Folic acid receptor overexpression observed in the breast, colon, ovary, kidney, or lung cancer cells [55] makes folic acid (FA) a popular targeting ligand. Wu et al. [33] reported DOX-loaded HNTs functionalized with poly(ethylene glycol) (PEG) and FA (DOX@HNTs-PEG-FA) as efficient carriers. HNTs, initially modified with (3-aminopropyl)triethoxysilane (APTES), were then coupled with NHS-PEG-COOH, and FA, resulting in final carrier material HNTs-PEG-FA. DOX was then loaded into the carrier based on physical adsorption. Results of both in vitro and in vivo studies showed the effective delivery of the cytostatic to the tumor cells with FA receptor overexpression, while no cytotoxic effect was observed for the normal cells. Furthermore, the DOX@HNTs-PEG-FA system showed no cytotoxicity against cells with negative FA receptor expression (L02 cell line), confirming its selectivity and the cytocompatibility of the carrier. Inhibited proliferation, induced apoptosis, caspase-3 cleavage, and Bcl-2 expression decrease were observed upon DOX@HNTs-PEG-FA interaction with MCF-7 and MCF-9 cell lines during in vitro studies. Tumor necrosis, with a significant reduction of its volume and weight, was observed after DOX@HNTs-PEG-FA intravenous administration to 4T1-bearing mice. Carrier-bound DOX had also a higher tendency to accumulate in cancer tissue, compared with normal tissues, leading to the decrease of systemic toxicity of the drug.

A slightly modified system for DOX delivery was shown by Mo et al. [56], in which HNTs were surface-modified with polyglycerol (PG). The modification resulted in a large number of hydroxyl groups on the surface of nanotubes, allowing for conjugation of more FA. Furthermore, the obtained system, Hal-PG-FA, was more stable in aqueous dispersions. DOX was incorporated into the carrier by incubating Hal-PG-FA in an aqueous solution of the drug [33]. It was shown that due to the presence of PG coating, DOX was loaded more effectively (31.6% compared to 18.6% for untreated HNTs), and its release was better controlled. The Hal-PG-FA nanocarrier was shown to be biocompatible toward both cancer (HeLa) and healthy (HMrSV5) cell lines. Incubation of the HMrSV5 cells with a DOX-loaded system (Hal-PG-FA/DOX) resulted in a nonsignificant cytotoxic effect, in comparison with a well-visible and dose-dependent decrease in viability of HeLa cancer cells. Cytotoxic effect was also significantly higher than that observed for a free DOX. Release studies showed that DOX was released much slower at normal physiological pH of 7.4 than in the acidic pH typical for the tumor site (6.2), as well as pH characteristic for endosomes (5.5–6.0) or lysosomes (4.5–5.0). Two possible cancer-targeting mechanisms were proposed by the authors based on these results: a passive accumulation of the carrier and successive DOX release in the acidic environment of the tumor, and active targeting by folic acid receptor-mediated transcytosis. All those facts suggest that Hal-PG-FA nanovehicles can be used to increase the therapeutic efficiency of anticancer agents targeting FA overexpressing tumors.

Grimes et al. [55] proposed a theragnostic, bifunctional HNTs-based system in which folic acid was used to target cancer cells, while fluorescein isothiocyanate (FITC) attached to the outer surface of HNTs, allowed for real-time monitoring of the delivery process. The functionalization procedure consisted of the HNTs’ reaction with N-[3(trimethoxysilyl)propyl]ethylenediamine (DAS) and further conjugation with FA. FITC was then attached to the surface of the nanotubes to obtain the final HNT-DAS-FA/FITC system. No cytotoxicity was observed for the CT-26 cancer cell line incubated with increasing doses of HNT-DAS-FA/FITC up to the concentration of 150 µg/mL, confirming the nanocarrier’s cytocompatibility. FITC present on the surface allowed to observe the successful and effective uptake of the nanovehicle in all concentrations studied and can be used in the future to verify the uptake of the system and the efficiency of the drug delivery. Finally, the presence of FITC and FA on the surface of HNTs decreased its tendency to aggregate, which was observed for nanotubes coated with LbL films, thus offering the alternative for such systems.

The very recent paper by Hu et al. [52] introduced a more advanced, theragnostic system in which the surface of the methotrexate-loaded HNTs was bifunctionalized with folic acid and fluorochrome. The folic acid presence increased the selectivity of the drug system toward osteosarcoma cells, while the modification of nanotubes’ surface with fotochrome allowed confirming the success of the cellular uptake of the system by cancer cells and facilitated the studies of the delivery mechanism. Modified, MTX-loaded HNTs were taken up by osteosarcoma cells via caveolae-mediated endocytosis. The system constructed in this way may be an attractive alternative delivery tool in osteosarcoma therapy. Luo et al. [57] performed additional studies on the carrier uptake and cellular death mechanisms of a similar system. Shortened HNTs used in that study were characterized by more uniform size distribution and higher cellular uptake. A higher cytotoxic effect was also observed for shortened HNTs, compared to regular-sized unmodified HNTs, the observation reported also by Liao et al. [53]. The uptake mechanism for the described bifunctional system was caveolae-mediated endocytosis triggered by the binding of the carrier to the cellular FA receptor. A significant amount of short HNTs accumulated near the plasma membrane, facilitating the uptake process. In vitro tests concerning selective targeting, conducted on colon cancer, osteosarcoma, and pre-osteoblast cell lines, revealed great osteosarcoma targeting efficiency combined with cell proliferation inhibition. The other two cell lines were not affected by the proposed system. These results suggest the excellent selectivity of the proposed drug delivery system. Cytotoxicity results showed a possibility of a significant reduction of MTX doses during osteosarcoma treatment, thus limiting the unwanted side effects.

Further modifications of HNTs were based on the exploiting of photophysical phenomena for more effective cancer therapy. This search led to the incorporation of metallic nanoparticles onto the HNTs surfaces [55]. Rao et al. [58] introduced chitosan-coated HNTs loaded with curcumin-Au hybrid nanoparticles for nontargeted delivery. Zhang et al. [45] proposed a novel HNTs-based nanoplatforms loaded with gold nanorods (GNRs) and DOX to target and eliminate breast cancer cells using FA ligands and NIR irradiation. The process of loading GNRs into HNTs’ lumen was achieved by using capillary forces in the mixture of HNTs and gold ions, followed by the addition of ascorbic acid. HNTs’ lumen was used as a template for nanorods formation. The surface of the resulting particles was then loaded with DOX, followed by an electrostatically driven process of coating with BSA. Further covalent binding of FA to BSA resulted in the Au-HNT-DOX@BSA-FA system. In vitro studies conducted for HNTs, Au-HNTs, and Au-HNT-DOX@BSA-FA on three cancer cell lines (MCF-7, HeLa, 4T1) and normal HUVEC cells confirmed the biocompatibility of Au-HNTs. Their photothermal activity resulted in cell apoptosis after NIR irradiation. Au-HNT-DOX@BSA-FA showed proapoptotic properties both without, and with laser irradiation. It was also shown that an increase in local temperature after irradiation promoted DOX release from the carrier. In vivo tests on mice demonstrated that Au-HNT-DOX@BSA-FA changed neither the shape nor the number of RBCs, white blood cells, or platelets. Furthermore, biochemical tests showed no toxic effect of Au-HNT-DOX@BSA-FA administration on the liver and kidney functioning. Studies on the photothermal effect of Au-HNT@BSA-FA and Au-HNT-DOX@BSA-FA carried out on mice bearing 4T1 showed no significant differences in the PTT effect in these two materials, confirming that DOX does not interfere with the photothermal effect. Application of Au-HNT@BSA-FA, followed by irradiation, caused a reduction of weight and volume of the tumor. This chemophotothermal nanoplatform significantly decreased DOX side effects and exceed the chemo- and photothermal therapy effects alone.

Another interesting approach, also focused on phototherapy of cancer, was presented by Li et al. [59]. Authors loaded HNTs with a photosensitizer, the indocyanine green (ICG), and coated them with poly(sodium-p-styrenosulfonate) (PSS) to increase their biocompatibility. The nanotubes were then further surface-coated with MDA-MB436 cell membranes to achieve the effective targeting of breast cancer cells via the Pickering effect. The proposed carrier system showed higher ICG encapsulation efficiency in comparison to the synthetic nanocarriers. It also prevented photochemical degradation of the dye and increased ICG’s stability in aqueous solutions. Additionally, HNT-PSS-ICG turned out to be a better photosensitizer and exhibited better photothermal properties than ICG alone, causing efficient photodynamic oxidative destruction of model GUV (giant unilamellar vesicle) membrane after NIR irradiation. Studies performed on the MDA-MB436 cell line revealed that HNT-PSS-ICG coated with the corresponding membrane tended to specifically target tested cancer cells, because of the presence of inherent antigens. Accumulation of HNT-PSS-ICG-MDA-MB436 in cancer cells was also confirmed, proving further the active targeting ability of this system. The tests performed in vivo showed a significant decrease in the tumor volume during the treatment of the tumor-bearing mice, contrary to the observed weak therapeutic effect after ICG and HNT-PSS-ICG administration. The authors suggested the main contribution of the photodynamic (PDT) effect rather than the photothermal (PTT) effect in the anticancer activity of the HNT-PSS-ICG-MDA-MB436 system in vivo.

The magnetic field may also be used for effective targeting of the nanocarrier–drug system to the cancer tissue. Various multimodal systems based on HNT modifications with iron oxides were reported for magnetic targeting in the literature. Guo et al. [42] presented HNTs tethered with Fe_3_O_4_ nanoparticles and functionalized with folic acid (FA-Fe3O4@HNTs) to deliver DOX electrostatically coupled to the carrier. The drug was released in a pH-specific manner due to the electrostatic nature of HNTs and DOX interactions. In pH = 5, found in cancer tissues, the drug release was significantly faster than in pH = 7.4, typical for normal tissues, allowing for the preferential delivery of DOX to a tumor site. Furthermore, in vitro MTT assay showed that DOX-loaded FA-Fe_3_O_4_@HNTs exhibited enhanced toxicity toward the HeLa cell line in comparison to the free drug. Another pH-responsive carrier was proposed for the targeted colon cancer therapy by Dramou et al. [28], where halloysite was used as a carrier of camptothecin (CPT). Magnetic HNTs coated with chitosan oligosaccharides (COS), functionalized with FA (FA-COS/MHNTs), and containing the adsorbed CPT were synthesized. Faster drug release was observed at pH = 5, in comparison to pH = 6.8 and 7.4, similarly to the system reported by Guo et al. [42]. Biocompatibility assay conducted on a blank (drug-free) FA-COS/MHNTs showed negligible cytotoxicity, which was explained by the presence of COS coating. Cytotoxicity for the CPT-containing system was slightly lower, compared to a free drug in the same concentration, which the authors attributed to a gradual release of CPT from the carrier material. The system with no FA attached exhibited a lower toxic effect than CPT@FA-COS/MHNTs toward the Caco-2 cells characterized by high expression of FA receptor. The proposed system was thus successful in actively targeting cells with overexpressed FA receptors and allowed the decrease in side effects of CPT administration. As mentioned in the previous section, Li et al. [50] developed a pH-responsive halloysite–polymer composite system for oral administration, which proved to be useful in targeted therapy of colon cancer. pH-responsive dissolution studies confirmed the pH-dependent dissolution of the carrier in pH above 6.8 since HPMCAS is not soluble in acidic conditions. Thus, the encapsulated drugs were protected from acidic stomach conditions and released after they reached the intestine and colon. The proposed formulation was less cytotoxic than a mixture of free drugs at pH = 6.5, while in physiological pH, it was more effective.

Using halloysite nanocontainers to maintain enzymatic activity in enzyme-based therapies was also reported. Min et al. [60] presented chitosan-coated HNTs decorated with Fe_3_O_4_ and antiproliferative enzyme laccase (Fe_3_O_4_-HNT-CS-Lac) for targeted delivery to estrogen-receptor-expressing cancer cells. Two-step synthesis consisted of the reductive precipitation of Fe_3_O_4_ on the surface of HNTs, followed by coating with chitosan and immobilization of laccase. In vitro studies on several human cell lines (liver HepG20, lung H460, stomach AGS, cervix HeLa) confirmed the preservation of the enzymatic activity of laccase, resulting in the antiproliferative effect and induction of apoptosis. For comparison, free laccase, while actively inhibiting the proliferation process, did not show any proapoptotic ability. The mechanism of carrier internalization was not fully studied, but the authors suggested Fe_3_O_4_-HNT-CS-Lac could be taken up by the cells by either endocytosis or receptor-mediated transcytosis. Massaro et al. [61] proposed a multivalent HNTs-based carrier functionalized with cyclodextrin (CD) derivative modified with pendant thiosaccharide chains, which allowed for exploiting lectin–carbohydrate interaction, which plays an important role in the cell recognition processes during tumor progression. β-CD was used to increase HNTs compatibility with aqueous media and to entrap an anticancer agent, curcumin, in a host–guest complex. The obtained composite allowed for the effective encapsulation of curcumin molecules and was proposed by authors as a promising targeted delivery system for various anticancer drugs. A few years later, Hu et al. [52] designed a multifunctional redox-responsive HNTs-based carrier with CD derivative and folic acid for targeted DOX delivery. HNTs modified with -SH groups were coupled with per-thiol-β-cyclodextrin cup and decorated with folic acid-polyethylene glycol-adamantane (FA-PEG-Ad) antenna through the complexation of β-CD with adamantane (Ad). DOX loaded into the HNTs lumen was released specifically in the presence of the overexpressed glutathione in tumor cells, because of the disulfide bond cleavage followed by the detachment of the whole β-CD-Ad-PEG-FA moiety. In vitro studies on cancerous SKOV3 cell lines, as well as the in vivo studies in SKOV3 tumor-bearing nude mice, supported the previously reported, excellent results [33,56] concerning FA-targeting delivery systems. The proposed HNTs carrier was characterized by selective targeting, good biocompatibility, efficient delivery, side effects reduction, and controlled release of the drug. Finally, the magnetic nanoparticles combined with CD and FA modified HNTs were utilized as a drug carrier by Li et al. [62]. The incorporation of magnetite into the outer surface of the halloysite enabled the conjugation of more CD-Ad-PEG-FA moieties, compared to the Hu et al. system [52]. The proposed system was designed for the isolation of tumor cells from the bloodstream for diagnostic or therapeutic purposes and will be discussed in detail in Section 5. The specific interactions of HNTs with cancer cells and subsequent examples of CTCs targeting by halloysite–nanocarriers are summarized in the next sections.

## 4. Interactions of HNTs and HNTs-Based Systems with Cancer Cells

One of the first steps when using a material in biomedical applications is to assess its biocompatibility with various cell lines. Thus, there are numerous studies on the biocompatibility of HNTs, many of them performed for various cancer cell lines, in order to evaluate the influence of halloysite nanotubes as carriers on the targeted tissues. Vegarro et al. [63] studied the cytotoxicity of various concentrations of HNTs on two different cancer cell lines: cervical adenocarcinoma (HeLa) and breast cancer (MCF-7) using two different assays, based on MTT and Trypan Bleu. Tests showed that adding as much as 75 μg/mL of HNTs to the culture did not decrease cells’ viability by more than 20%. The uptake studies performed using fluorescently labeled HNTs, modified with APTES and FITC, showed that HNTs penetrate both cell types easily and locate in the vicinity of nuclei but do not negatively influence cell proliferation. Kamalieva et al. [64] studied the HNTs’ biocompatibility with adenocarcinoma epithelial cells (A549), a popular cancer cell model extensively used in drug delivery research. The cytotoxicity results were similar to previous studies with a 75 μg/mL concentration of HNTs as a limit for nontoxicity of HNTs. The authors have also estimated the 50% inhibitory concentration as 300 μg per 105 cells and confirmed the dose-dependent reduction in cell growth. Importantly, it was shown that the application of different colorimetric viability tests (LDH, MTT, rezurcin test, neutral red test) may give different results—in this case, LDH enzymatic test differed significantly. The authors concluded that it is always important to verify colorimetric tests with other independent assays, e.g., confluence observation using optical microscopy. Combined enhanced darkfield microscopy, TEM, and AFM allowed confirming without a doubt that HNTs do not penetrate the cell’s nuclei. Liu et al. [65] performed cytotoxicity evaluation of HNTs in the A549 cell line complemented with the hemocompatibility studies. Three different hemocompatibility tests were used to evaluate HNTs: hemolysis ratio test, plasma clotting activity assay, and platelet activation process. The hemolysis ratio was below 5% for the sample concentrations up to 200 µg/mL, confirming the full safety of HNTs in contact with the red cells. Procoagulant activity and significant reduction of clotting time were, however, obtained for the HNTs’ samples of concentrations exceeding 50 µg/mL. This result correlated well with the significant platelet activation and aggregation in the higher (≥50 µg/mL) HNTs concentrations. The authors pointed out the 50 µg/mL concentration as a limit for the nontoxicity of the nanotubes toward A549 cells; however, the cell viability was still at 75% for HNTs concentrations up to 100 µg/mL after 72 h. TUNNEL assay allowed to observe massive DNS breaking, a sign of late apoptosis, in A549 cells at HNTs concentrations as high as 50 µg/mL. Liu et al. [66] recently studied the possible mechanism of HNTs internalization and transport inside A549 cells. The two most probable routes of HNTs internalization were studied: clathrin-dependent endocytosis and caveolae-dependent endocytosis, and the authors concluded that both pathways are involved. The authors also tracked fluorescently labeled HNTs inside the cells and confirmed that the transport occurs through microtubules and actin filaments, and HNTs colocalize with both: lysosomes and Goldi apparatuses.

Several studies performed for pristine HNTs in vivo conveyed their influence on nonmammalian organisms. Fakhrullina et al. [67] studied the influence of HNTs on the nematode Caenorhabditis elegans in order to evaluate the potential harm risk of industrially used halloysite on the environment. The authors found that HNTs were safe for Caenorhabditis elegans at the concentration as high as 1 mg/mL, which is much higher than the possible soil contamination concentrations. Long et al. [68] studied the influence of HNTs on zebrafish embryos and larvae. The cytotoxicity of different concentrations of HNT nanotubes was evaluated, as well as the biodistribution of HNTs in these organisms. No acute or lethal toxicity was observed for the HNTs concentrations lower than 25 mg/mL. HNTs were shown to accumulate on the surface of the chorion, which is a protecting barrier of the embryo. Once the concentration reached or exceeded 25 mg/mL, the nanotubes wrapped fully over the surface of the chorion, promoting the hatching of zebrafish. It was also shown that HNTs can be ingested and then excreted by zebrafish larvae at concentrations up to 25 mg/mL. The in vivo studies in mice showed toxicity and pulmonary fibrosis at the dose of 50 mg/kg of body weight, while the dose of 5 mg/kg of body weight given for 30 days proved to be safe [69]

Very often, instead of simply load the drug inside the halloysite nanotubes, the practice is to modify their surface with various polymers, either to enhance their stability in aqueous media or to functionalize their surface further (e.g., for targeted therapy). One of the polymers the most extensively used for these modifications is PEG. Chiriaco et al. [70] coated the surface of HNTs with covalently attached PEG, and cytotoxicity of the obtained HNTs-PEG system was evaluated in two different cell lines: MCF-7 and HeLa. PEG-coated HNTs were shown to be fully biocompatible for both cell lines at concentrations up to 100 µg/mL, with close to 100% viability of the cells after 24 h, and still above 70% viability after 72 h of incubation with HNTs-PEG.

A very interesting study aimed at comparing the cytotoxicity of HNTs, Fe_3_O_4_ nanoparticles (Fe_3_O_4_ NP), and HNTs/Fe_3_O_4_ NP nanocomposite toward bacteria, normal cells, and cancer cells was conducted by Abhinaya et al. [71]. They showed that HNTs reduced the cytotoxicity of iron oxide nanoparticles toward bacteria by changing the hydrophilicity, size, and surface charge of the material. Another part of the study was conducted for the noncancerous Vero cell line and human lung cancer cell line A-549. While for Vero cells both Fe_3_O_4_ NP and HNTS/Fe_3_O_4_ NP were found biocompatible, the nanocomposite proved to be more cytotoxic toward A-549 than Fe_3_O_4_ NP alone. Thus, the nanocomposite showed specific, significant cytotoxicity to cancerous cells while being biocompatible to normal cells and bacteria. This indicates that cancer cells are specifically sensitive to HNTs/Fe_3_O_4_ NP nanocomposite, which makes it a promising material in cancer therapy. The toxicity of the HNT/Fe_3_O_4_ nanocomposite can be thus attributed to the cell type and cell specificity.

HNTs were also used to deliver small interfering RNA (siRNA) to alter the processes inside the cancer cells. siRNA prevents translation of the genes with complementary nucleotide sequences by causing the degradation of mRNA after transcription. A variety of siRNA sequences were designed to target the oncogenes involved in carcinogenesis, or for silencing target molecules crucial for tumor–host interactions, or the resistance to chemotherapy or radiotherapy. siRNA, being ionic, cannot enter the cell via diffusion. It is also rapidly removed from the bloodstream by enzymatic digestion and renal elimination, thus requiring an appropriate delivery system. HNTs were used to deliver siRNA, with suitable surface modifications facilitating transfection. APTES and poly(ethylene imine) (PEI) were used for this purpose [72,73]. Long et al. [74] proposed polyamidoamine dendrimer grafted halloysite nanotubes (PAMAM-g-HNTs) loaded with siRNA for breast cancer therapy. The nanovehicle showed good biocompatibility with HUVEC and MCF-7 cell lines in a concentration range up to 100 μg/mL, while siRNA-loaded system delivery resulted in the significant apoptosis of MCF-7, higher than that observed for a well-known Lipofectamine 2000 system. These results were confirmed also in vivo, in 4T1-bearing mice. Liu et al. [75] used HNTs to deliver siRNA sequence actively targeting the RIPK4 oncogene, which is overexpressed in skin, ovarian, cervical, and colorectal cancers. To investigate the cytotoxicity of the carrier system, the negative control siRNA (siNC) was complexed with HNTs and incubated with T24 cells originating from a malignant human urinary bladder carcinoma. Even at high concentrations, the HNTs/siNC complex did not significantly affect cell viability. HNTs delivered siRIPK4 sequence efficiently and specifically to bladder cancer cells, achieving targeted gene silencing, inhibiting bladder cancer growth both in vitro and in vivo.

In addition to siRNA, ribonucleases (RNases) have also been proposed in anticancer therapy since they may destroy RNA in cancer cells and produce signals leading to cancer cells’ programmed death. Khodzhaeva et al. [76] immobilized Bacillus pumilus RNase-binase-on HNTs. Cytotoxicity studies toward human colon adenocarcinoma Colo320 confirmed that while the cells’ viability after 24 h incubation with pristine HNTs was as high as 97%, the free binase and binase-loaded HNTs reduced the number of viable cells by 27% and 64%, respectively. These results confirmed the huge potential of HNTs/RNase systems in cancer therapy.

**Figure 3:**
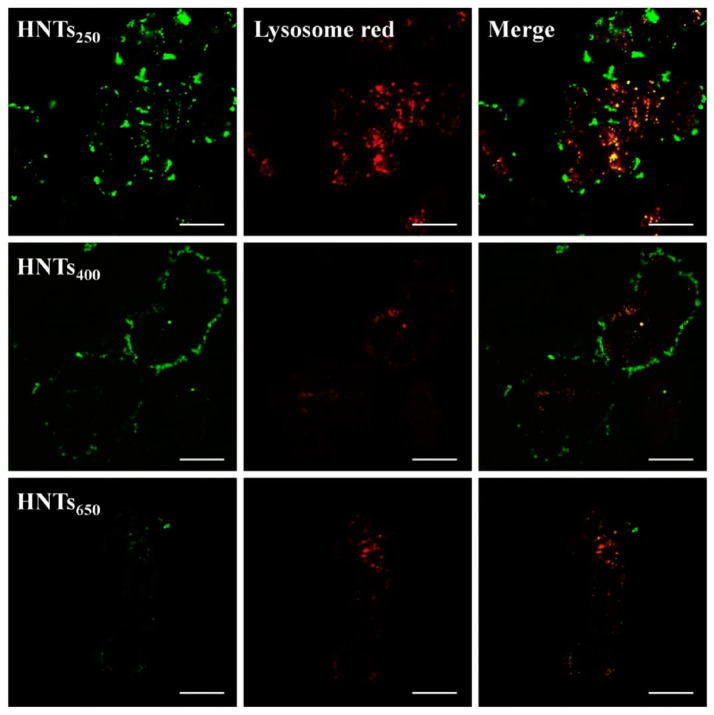
Intracellular uptake and subcellular distribution of HNTs_250_, HNTs_400_, and HNTs_650_ with HT-29 cells. Scale bar = 25 μm. Reprinted from *Colloids and Surfaces A*, Vol. 585, Juan Liao, Siyu Peng, Mei Long, Yaya Zhang, Huaming Yang, Yi Zhang, Jing Huang, Nano-Bio interactions of clay nanotubes with colon cancer cells, No. 124242, Copyright (2020), with permission from Elsevier.

The size of the nanotubes may have an important influence on their cytotoxicity, cellular internalization, as well as localization and distribution within the cell. Pristine halloysite nanotubes have a size of ca. 1–1.5 µm and relatively large polydispersity. To downsize and unify the length of the HNTs they may be pretreated by a high-speed shear homogenization/ultrasonic scission and fractionated through the two-step uniform viscosity centrifugation, as reported in the literature [77]. Liao et al. [53] studied shortened HNTs of three different sizes, obtained by ultrasound scission combined with viscosity and density gradient centrifugation. The HNT diameters measured by DLS were 275.4 nm (HNTs_250_), 413.8 nm (HNTs_400_), and 665.3 nm (HNTs_650_). They studied HNTs’ biodistribution and cellular uptake in the HT-29 (human colon cancer) cell line. For all the sizes tested, the cell viability measured by the MTT test was above 77% after 24 h and still above 74% after 72 h of incubation, even at HNTs’ concentrations up to 500 μg/mL. Thus, no significant influence of the nanotubes’ size on their cytotoxicity was detected. Cellular uptake studies showed that the internalization mechanism consists of the three phases: adsorptive endocytosis, exocytosis, and balance. Figure 3 shows CLSM results, in which lysosomes were labeled with Lyso-Tracker Red and HNTs with FITC (green fluorescence); they indicate that the FITC fluorescence intensity was positively correlated with the concentration of internalized particles. HNTs were taken up by cells and accumulated in lysosomes, as demonstrated by the presence of yellow fluorescence on the merged images. The FITC-related fluorescence intensity decreased with increasing size of HNTs. CLSM, as well as TEM and flow cytometry measurements, revealed that the smallest HNTs_250_ showed increased uptake, in comparison to HNTs_400_ and HNTs_650_.

Luo et al. [57] prepared the bifunctional HNTs modified with two ligands: folic acid and fluorochrome and, among others, studied the influence of the size of such targeted nanotubes on their cellular interactions. The commercially available HNTs (0.914 nm) were shortened by ultrasonication to obtain the smaller nanoparticles (0.715 nm). HNTs of both sizes were then bifunctionalized and their interactions with cells studied. As estimated for CT26WT (murine colorectal cancer) cell line by flow cytometry, for the commercial HNTs 20% of cell death was caused by apoptosis and 10% by necrosis. For the shortened nanotubes, the apoptosis- and necrosis-related cell deaths were at the level of 23% and 13%, respectively. The authors concluded that in both cases cell death was mainly due to apoptosis. Shortened bi-HNTs resulted in overall higher cell death, most probably due to the increased accumulation inside the cells. These studies have also confirmed the caveolae-mediated endocytosis as the main uptake pathway of the proposed bifunctional HNTs.

In vivo studies on the HNTs are very limited. In nematode Caenorhabditis elegans, the uptake of HNTs by intestinal epithelial cells was very low; thus, the ingestion of HNTs in a broad range of concentrations up to 1 mg/mL was found to be safe [67]. HNTs have also shown no acute toxicity in a zebrafish toxicity model and thus were claimed to be environmentally safe at concentrations lower than 25 mg/mL [68]. Recently, the toxicity of HNTs after oral administration, inhalation, and intratracheal instillation was evaluated in murine in vivo models. Wang et al. [78] have recently shown that HNTs may induce Al accumulation after 30-day repeated oral administration. Oral application of HNTs at 5 mg/kg BW stimulated mice growth and caused no pulmonary toxicity. A higher dose of 50 mg/kg BW HNTs inhibited mice growth and resulted in lung inflammation and pulmonary fibrosis induction.

Finally, Liu et al. [75] studied in vivo the effect of HNTs as delivery systems for RIPK4 siRNA in the therapy of bladder cancer. The authors chose HNTs as the best vehicle for RIPK4 siRNA since they were able to transfect selectively bladder cancer cells in vitro. HNTs were shown to indeed passively target and accumulate in bladder tumors, unlike, e.g., liposomes or polymer nanoparticles. The authors proposed that the HNTs/RIPK4 siRNA complex can cross the hepatic sinusoidal endothelium and glomerular filtration barrier and is able to access the malignant tissue. The HNTs/siRIPK4 complexes showed no apparent organ toxicity and the functional parameters of the liver and the kidney were well within the norm.

## 5. The Application of HNTs in Cancer Cell Capture

Circulating tumor cells (CTCs) are shed from the primary tumor during its formation and growth to the bloodstream. They may stay dormant for years after the primary tumor was completely removed and eventually cause metastasis, i.e., they form a secondary tumor in the distant organ. However, to do that, CTCs must migrate to the distant organ, extravasate, invade the surrounding tissue, adhere, and start to proliferate. Metastasis is responsible for ca. 90% of all cancer-related deaths; therefore, early detection and removal of CTCs from the bloodstream of the patients diagnosed with cancer are the focus of intense studies. Different approaches have been proposed allowing to capture CTCs from the bloodstream, all trying to overcome the most important obstacles—the extremely low concentration of CTCs in the blood, in comparison to other blood elements, and CTCs’ heterogeneity [79].

**Figure 4:**
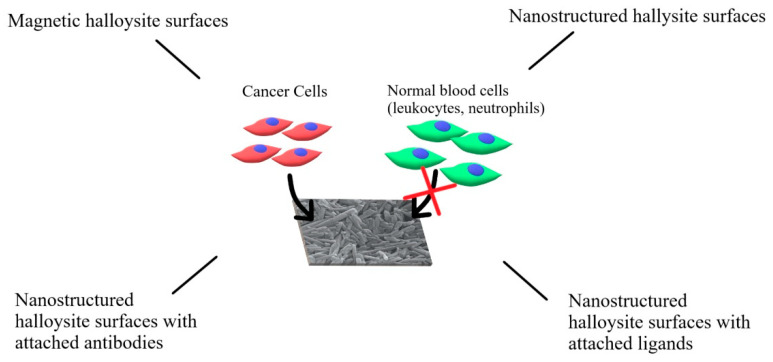
Various types of HNT-based surfaces designed to capture CTC. HNTs surfaces effectively capture cancer cells but not normal blood cells.

HNTs can be used to enhance and guide the interactions with cancer cells, especially with CTCs, by forming nanostructured surfaces. Such surfaces can be used to engineer various systems designed to capture CTCs for diagnostic or therapeutic purposes. Several approaches were taken, starting from the simple patterned or rough surfaces made of HNTs, through the nanostructured HNT coatings modified with appropriate targeting molecules (antibodies or ligands), to the drug-loaded coatings designed to capture and kill CTCs, and magnetic HNT surfaces (see Figure 4). To obtain such coating, HNTs are typically pretreated to decrease and unify their size—this is usually achieved using either a sonic disintegrator or simply a mortar. Then, HNTs are suspended in an aqueous medium and used to coat the surface.

The studies on the application of the HNTs surfaces in cancer treatment started a decade ago. Hughes et al. [41] utilized halloysite to improve their CTCs capture system based on the highly blood-compatible, polyurethane Micro-Renathane^®^ microtubing surface-modified with P-selectin and E-selectin. Such a system was a model for inflamed endothelium, where selectins are expressed on the luminal surface in order to interact with leukocytes, which first are weakly bound, then, through a rolling motion, interact further, adhere firmly, and enter the tissue [80]. A similar mechanism was described for extravasating CTCs [81]. The incorporation of the HNTs’ coating with selectins bound on it allowed the significant enhancement of the performance of the system. HNTs’ coating was introduced through electrostatic interactions with the poly-L-lysine interlayer. The presence of rough nanostructured HNTs layer allowed to significantly reduce the rolling velocity of the model cancer cells (acute myeloid leukemia KG1a line), as well as increase the number of captured cells. The proposed explanation is that some nanotubes are “sticking out” above the surface and thus may interact with cells that are suspended further from the surface of the microtube. Authors have also shown that HNTs do not influence the viability of KG1a cells. As an additional profit, more P-selectin adsorbed on HNTs than on the plain surface of the microtubes. The interesting, complementary studies conducted by the same group proposed to utilize the CTCs capture system described above to perform a personalized sensitivity test for the patient, designed to find the most effective chemotherapeutic in order to eliminate CTCs [82]. The idea was to divide the blood sample of the metastatic patient into multiple aliquots and add a different chemotherapeutic to each of them. From another sample of the same patient, CTCs were isolated and the same number of them was added to each aliquot. After incubation, each aliquot was run through the CTCs capture system and the number of living CTC was calculated in each of them. The reduction in the number of live CTC cells was a proposed measure of sensitivity to each drug. The main advantage of that approach was the possibility of testing chemotherapeutics in the whole blood and in the flow, similar to the in vivo conditions. Some unwanted cell death due to the necessary surface blocking with BSA was, however, noticed. The Hughes group also compared the proposed E-selectin modified microtube system with the FDA-cleared commercial CellSearch^®^ method [83]. Out of twelve samples processed via CellSearch^®^, five were found negative for CTCs, while all these same five patients were found positive with the system proposed by Hughes, thus showing it had a better sensitivity. This finding was also confirmed by the significantly higher yield of CTCs captured by the E-selectin-functionalized device.

At the same time, in 2012, Mitchell et al. [46] published a study showing that, unlike in the case of CTCs, the adhesion of the normal cells (neutrophils) to the microrhenathane^®^ microtubing was significantly reduced by the presence of HNTs nanostructured coating. Another advancement was that instead of E-selectin, they used DOX-loaded, E-selectin-functionalized liposomes, which were attached either to the smooth walls of the microtubes or to the surface of HNTs covering microtube’s walls. Much more cancer cells (MCF7, COLO 250) were captured on the HNT coating, confirming the observation of Hughes [41]. The opposite effect was observed for neutrophils–their adhesion was prevented by the presence of halloysite. Due to the DOX loading, the cancer cells were not only captured but also killed. It was shown that the HNTs’ coating increased both the capture and the kill rates of cancer cells in the flow conditions. These conclusions were again confirmed by Hughes et al. [84] in the E-selectin-functionalized system. On the smooth E-selectin-modified microtubes, leukocytes spread and adhered well, while this process was prevented by the presence of the optimized halloysite coating prepared using suspensions containing 0.4 to 0.8% of HNTs (studies performed for the whole blood samples). The authors explained the disparity in the interactions of cancer cells and leukocytes with HNT coating as caused by the differences in the distribution of selectins on the surface of those cell lines. It was established, based on microscopic observations, and confirmed by molecular modeling that lateral spacing of the surface features, rather than its overall roughness, was a decisive factor in those interactions. Mitchell group also verified the possibility of modifying HNTs coating with surfactants prior to E-selectin adsorption [85]. Sodium dodecanoate (NaL) was shown to increase the roughness of HNT coating, as well as the amount of adsorbed E-selectin, while further preventing leukocyte capture. Surprisingly, unlike anionic surfactants, decyltrimethylammonium bromide (DTAB) generated the opposite cell response: an enhancement in leukocyte and decrease in cancer cell capture.

Further development was preceded by the studies conducted on the controlled processes of the self-assembly of HNTs into the organized structures. He et al. [86] prepared patterned HNT coatings on the glass substrate by thermal drying. HNTs were first modified by adsorbing poly(styrene sulfonate) (PSS) on their surface. Then, a stable suspension of such nanotubes was injected into the slid made between two glass slides and dried at 60 °C. That resulted in the self-assembly of HNTs into the coating with ordered arrays of nanotubes and regular cracks. The microstructure of the coating could be fine-tuned through the right choice of the HNTs concentration in suspension. It was found that the roughness of HNT coating prepared using 10% HNT suspension was very similar to the surfaces made of TiO_2_ nanoparticles on a glass substrate, which were previously used to capture CTCs [87]. An HNTs-coated surface obtained using 2% suspension had the highest cell capture rate: after incubation for 3 h, around 80% of cancer cells (MCF-7, HepG2, Nero-2a, A549, and B16F10) and ca. 27% of control (normal) cells (MC3T3-E1 and L02) were captured. The authors suggested that the rough HNTs coating increased significantly substate–cell interactions and pointed out the different surface structures of cancerous and normal cells. They also found out that the cells were more flattened on the HNTs-patterned coating. Such a system was subsequently surface-modified with streptavidin, and a biotinylated anti-human EpCAM/TROP1 antibody was attached. Again, the functionalization significantly increased the efficiency of capture of the cancer cells (from 80% to 92% for MCF-7 cell line). Additional tests conducted in spiked blood confirmed a very low capture rate of white cells (0.01–0.025%) and a high capture rate of cancer cells (80%) after 3 h of incubation. An interesting review on interfacial self-assembly of HNTs was proposed by Lvov et al. [88], including also patterned surfaces for cell capture and orientation.

**Figure 5:**
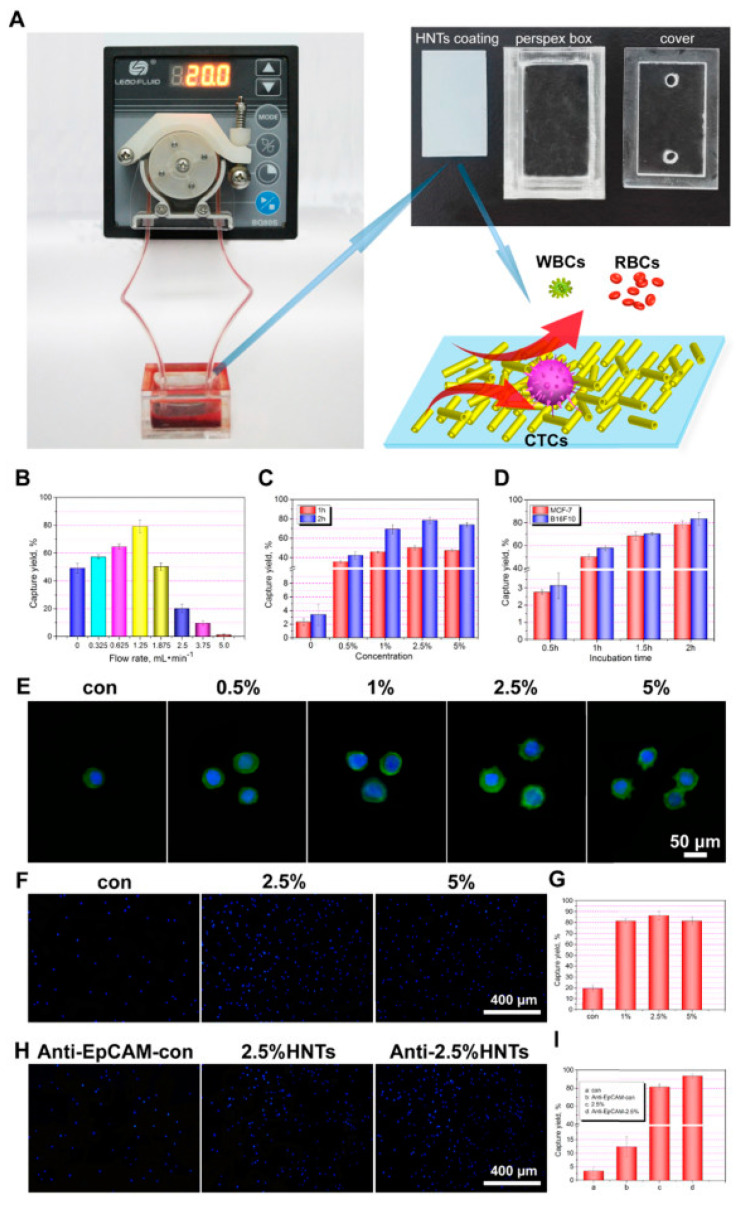
The photo of a circulating device with a peristaltic pump and the schematic diagram of cell capture from whole blood (**A**). The capture yield to MCF-7 cells with different flow rate (**B**). The capture yield to MCF-7 cells with different rough HNTs coating surfaces (**C**). The capture yield to MCF-7 cells at different times (**D**). The Alex Flour 488 and DAPI stained fluorescence microscopy images of captured cells of MCF-7 on the different coatings for 2 h (**E**). The DAPI stained fluorescence microscopy images showing that the MCF-7 cells captured by different coating surfaces under dynamic shear conditions for 1 h and then standing for another 2 h (**F**) and quantification of the captured MCF-7 (**G**). The DAPI stained fluorescence microscopy images of the MCF-7 cells captured by smooth glass and 2.5% HNT coating without and with anti-EpCAM conjugation for 2 h at a flow rate of 1.25 mL·min^−1^ (**H**) and the cell capture yield (**I**). Reprinted from *Materials Science & Engineering C*, Vol 85, Rui He, Mingxian Liu, Yan Shen, Rong Liang, Wei Liu, Changren Zhou, Simple fabrication of rough halloysite nanotubes coatings by thermal spraying for high performance tumor cells capture, Pages 170–181., Copyright (2018), with permission from Elsevier [47].

All the previous studies were carried out on relatively small surfaces, which limited their possible application. Another milestone consisted of the new, fast and cost-effective methodology to obtain large-area HNT-coated surfaces via thermal spraying of HNTs ethanolic dispersions proposed by He et al. [47]. HNTs in the ethanolic dispersion were sprayed from the nozzles of an airbrush under air pressure on hot glass. Ethanol was evaporated, and HNTs were bound on the surface through van der Waals forces and hydrogen bonds. The obtained coatings were transparent when obtained from the dispersions containing below 1% of the HNTs and semitransparent even for 5% dispersions. The authors tested a series of different conditions and came up with the optimal ones. The maximum cancer cells capture yield was obtained with the HNTs coating obtained by thermal spraying of 2.5% ethanolic suspension of the nanotubes, modified with an anti-EpCAM antibody. Such a system was incubated for 2 h with the sample containing cancer cells by using a 1.25 mL/min flow rate with a peristaltic pump. These conditions allowed capturing 93% of the MCF-7 cells from the artificial blood samples spiked with MCF-7 cells and gave similarly good results with other cancer lines (HepG2, A549, PC3, B16F10) but not for HeLa cells. The latter can be explained by the difference in the cell surface structure. Both the system and the results are presented in Figure 5. The authors next immobilized DOX by adsorbing it onto the surface of HNTs. The obtained loading of DOX was 0.4%. No influence of DOX on cancer cells’ capture was observed. The viability of cancer cells on such coating was then evaluated at different times. At first, the viability of MCF-7 cells was only 10% lower for HNTs-DOX than for HNT coating, but after 4 h, the difference was already in the range of 50%, and after 16 h, the survival of MCF-7 on the HNTs-DOX coating was decreased by 100%. MCF-7 cells died on this coating after ca. 8 h. Anti-EpCM-antibody-modified HNTs-DOX coating exhibited a cancer cells capture efficiency similar to the CellSearch^®^ device, but importantly, it was also able to kill these cells due to the DOX release.

Several papers have recently reported magnetic HNTs systems, in which halloysite nanotubes were modified using iron oxide, either by surface modification with superparamagnetic iron oxide nanoparticles (SPION) [89,90], by incorporating these particles inside the lumen of HNTs [91], or by forming nanorods inside the lumen [92]. A magnetic HNTs-based system was also proposed for cell capture by Li et al. [62]. SPION were synthetized on the outer surface of HNTs using a typical coprecipitation method from iron (II) and iron (III) salts in alkaline conditions. The surface of nanotubes was then modified using APTES to introduce amine groups, and carboxylated β-cyclodextrin was covalently bound to the surface via an amide bond. In order to target cancer cells, folic acid was then introduced on the HNTs surface using adamantine as the “docking” molecule, able to form complexes with β-cyclodextrin and poly(ethylene glycol) chain as a linker, resulting in MHNTs@β-CD@Ad-PEG-FA material. Skov3, HeLa, and A549 cancer cell lines were used to test the efficiency of this new capture system, and HEK293T cells were used as a reference cell line. The viability of all four cell lines after incubation with the proposed composite was at ca. 88%, suggesting good biocompatibility of the system. Similar results were obtained for the carrier without FA units attached. Cellular uptake tests showed significantly better uptake of FA-bearing system by cancer cells. Further assessment of FA-receptor selective binding, performed on HEK 293 T cells, indicated specific targeting ability of MHNTs@β-CD@Ad-PEG-FA to cells overexpressing FA receptors. The capture efficiency of the system increased in a dose-dependent manner with increasing amount of surface modified HNTs, and for the optimal concentration range of 0.25–0.35 mg/mL, around 96% of cancer cells were captured, while for reference cell line, the efficiency was below 10%. The presence of SPION allowed magnetically separating the HNT-based system in stationary conditions. The proposed system was characterized by high survival rate of cancer cells, which can be beneficial for diagnostic purposes but not so much in therapeutic applications. The proposed capture method was characterized by high sensitivity and selectivity and it was confirmed that the same HNTs could be used many times since the capture efficiency decreased only about 3% per the subsequent three cycles. Such a system may constitute an attractive alternative for the HNTs-modified surfaces as a capture device, but its application in a flow conditions is yet to be developed.

## 6. Conclusions and Future Prospect

HNTs, even though known for years, are becoming an important material in biomedical applications. While they are considered to be biocompatible, only low concentrations have been proved to be safe for the body. Thus, additional studies are needed for them to be applied widely in drug delivery on the clinical level. On the other hand, HNTs have unique structures and properties, which can be used with success in cancer therapies, in which the tolerance for the side effects is often slightly higher and secondary to the effectiveness of the treatment. This is further supported by the abundant literature on the application of carbon nanotubes in the delivery of chemotherapeutics [93,94]. Carbon nanotubes (CNTs) share many properties with HNTs, including tubular shape, nanometric size, multilayer structure (multiwall CNTs–MWCNTs), and ability to entrap drugs. A number of anticancer drugs were effectively delivered to cancer cells using CNTs, including DOX [95,96], paclitaxel [97], methotrexate [98], and siRNA [99]. The advantages of the nanotubular multiwalled structure are often mentioned as the main advantage of the proposed CNT-based systems. HNTs with similar structure, shape, and size, and higher biocompatibility seem to be even better suited for the application. Hartwig et al. [100] have compared the efficacy of the recombinant LipL32 antigen delivery using MWCNTs and HNTs. The former had to be additionally surface-modified with carboxyl groups to reach sufficient compatibility with aqueous media [101,102], which is a well-known disadvantage of CNTs. Due to the biocompatibility considerations, MWCNTs were also used in the five times lower concentrations than HNTs, and it is known that single-wall CNTs (SWCNTs) are characterized by even higher toxicity [103]. Both systems proved to be suitable for antigen delivery.

Among the most exciting and promising applications of HNTs in cancer-related therapies are targeted systems containing siRNA and RNases complexed with HNTs, as well as magnetically modified, nanostructured HNT surfaces for effective capture of CTC. HNT coatings show a unique property of promoting the capture of various cancer cell lines while preventing the interactions with normal cells, mainly blood cells such as neutrophils or leukocytes. HNTs lumen and multilayer structure allow for the simultaneous entrapment and further delivery of two different anticancer drugs, even with different physicochemical characteristics, thus providing the means to capture and effectively kill cancer cells. Studied for only a decade, HNT-containing systems for CTC capture and for protein, siRNA, and gene delivery have a great prospect and should soon become a key component of the clinically tested and approved anticancer therapies.

## Figures and Tables

**Table 1:** A summary of the literature reports on anticancer drugs entrapped in HNTs.

Anti-Cancer Drug	Cancer Type	Drug Loading Method	HNTs Modification	Therapeutic Effect	References
Doxorubicin	Breast cancer	Immersion method	HNTs surface-modified with folic acid (FA) through PEG linker	Inhibited proliferation and induced death of MFC-7 cells	[33]
Doxorubicin	Tumors with overexpression of FA receptor	Immersion method	HNTs conjugated with magnetic particles (Fe_3_O_4_) and FA	Enhanced toxicity to HeLa cells	[42]
Doxorubicin	Breast cancer	Immersion method	HNTs grafted with chitosan oligosaccharide (COS)	Inhibited proliferation and induced death of MFC-7 cells	[44]
Doxorubicin	Acute myeloid leukemia	Immersion method	Loading HNTs with gold nanorods (GNRs) and conjugating them with FA via reaction. with bovine serum albumin (BSA).	Inhibited proliferation and induced death of MFC-7 cells, decreased drug toxicity	[45]
	Cervical cancer Colon cancer Hepatocellular carcinoma	Immersion method	HNTs immobilized with drug-loaded liposomes surface-modified with E-selectin	Increased number of MCF7 and COLO 205 cells captured	[46]
	Lung cancer Prostate cancer Melanoma	Immersion method	HNTs coated by thermal spraying of DOX loaded HNTs ethanol dispersion and conjugated with anti-EpCAM antibody	Enhanced toxicity to HeLa cells	[47]
Curcumin	Breast cancer Acute myeloid leukemia	Drug covalently attached to the HNTs surface	Microwave-assisted synthesis of HNTs conjugated with curcumin- HNTs modified with 3-Aminopropyltriethoxysilane (APTES) or 3-Glycidoxypropyltrimethoxysilane (GPTMS) by electrospinning	Enhanced toxicity to SUM 149, MCF-7, and MDA-MB-231 breast cancer cell lines also to HL60 and HL60R myeloid leukemia cell lines	[48,49]
Resveratrol	Breast cancer	Vaccume pump method	HNTs were coated with polyelectrolytes (PAH or PRM and PSS or DXS) using LbL method	Restrained proliferation and induced death of MFC-7 cells	[43]
Celecoxib	Colon cancer	Immersion method	HNTs modified with pH-responsive microspheres	Inhibited proliferation of colon cancer cells	[50]
Atorvastatin	Colon cancer	Immersion method	HNTs modified with pH-responsive microspheres	Inhibited proliferation of colon cancer cells	[50]
Methotrexate	Osteosarcoma	Immersion method	no further modifications	Induced apoptosis and significantly inhibited proliferation of cancer cells	[51,52]
Artemisinin	Osteosarcoma	Immersion method	no further modifications	Induced apoptosis and significantly inhibited proliferation of cancer cells	[51]
Quercetin	Osteosarcoma	Immersion method	no further modifications	Induced apoptosis and significantly inhibited proliferation of cancer cells	[51]
Taurolidine	Osteosarcoma	Immersion method	no further modifications	Induced apoptosis and significantly inhibited proliferation of cancer cells	[51]
Camptothecin	Colon cancer	Surface attachment of the drug	HNTs modified with magnetic particles (FeCl_3_∙6H_2_O and FeSO_4_∙7H_2_O) then conjugated with COS, Camptothecin (CPT), and FA	Induced apoptosis of Caco-2 cancer cells	[28]

## Data Availability

Data sharing is not applicable to this article.
